# Development and implementation of the International AIDA Network Castleman’s disease registry

**DOI:** 10.3389/fmed.2025.1579182

**Published:** 2025-09-03

**Authors:** Antonio Vitale, Jessica Sbalchiero, Valeria Caggiano, Stefano Lazzi, Samar Tharwat, Lampros Fotis, Giuseppe Lopalco, Giacomo Emmi, Piero Ruscitti, Abdurrahman Tufan, Gaafar Ragab, Pravin Hissaria, Lorenzo Dagna, Ewa Wiesik-Szewczyk, José Hernández-Rodríguez, Andres González-Garcia, Anastasios Karamanakos, Ezgi Deniz Batu, Haner Direskeneli, Florenzo Iannone, Ilenia Di Cola, Hamit Kucuk, Rahime Duran, Moustafa Ali Saad, Mahmoud Ghanema, Mariusz Sikora, Fatma Alibaz-Öner, Mark Beecher, Seza Ozen, Emanuele Cencini, Monica Bocchia, Alberto Balistreri, Claudia Fabiani, Bruno Frediani, Luca Cantarini, Pierluigi Zinzani

**Affiliations:** ^1^Research Center of Systemic Autoinflammatory Diseases and Behçet’s Disease Clinic, Department of Medical Sciences, Surgery and Neurosciences, University of Siena, Siena, Italy; ^2^Azienda Ospedaliero-Universitaria Senese [European Reference Network (ERN) for Rare Immunodeficiency, Autoinflammatory and Autoimmune Diseases (RITA) Center], Siena, Italy; ^3^Department of Medical Biotechnology, Institute of Pathology, Azienda Ospedaliero-Universitaria Senese, Siena, Italy; ^4^Rheumatology and Immunology Unit, Department of Internal Medicine, Faculty of Medicine, Mansoura University, Mansoura, Egypt; ^5^Department of Internal Medicine, Faculty of Medicine, Horus University, New Damietta, Egypt; ^6^Department of Pediatrics, Attikon General Hospital, National and Kapodistrian University of Athens, Athens, Greece; ^7^Department of Precision and Regenerative Medicine and Ionian Area (DiMePRe-J), Policlinic Hospital, University of Bari, Bari, Italy; ^8^Department of Medical, Surgical and Health Sciences, University of Trieste, Clinical Medicine and Rheumatology Unit, Cattinara University Hospital, Trieste, Italy; ^9^Centre for Inflammatory Diseases, Department of Medicine Monash Medical Centre, Melbourne, NSW, Australia; ^10^Department of Biotechnological and Applied Clinical Sciences, University of L’Aquila, L’Aquila, Italy; ^11^Division of Rheumatology, Department of Internal Medicine, Gazi University Hospital, Ankara, Türkiye; ^12^Rheumatology and Clinical Immunology Unit, Department of Internal Medicine, Faculty of Medicine, Cairo University, Giza, Egypt; ^13^Faculty of Medicine, Newgiza University, 6th of October City, Egypt; ^14^Department of Clinical Immunology and Allergy, Royal Adelaide Hospital, Adelaide, SA, Australia; ^15^Faculty of Medicine, Università Vita-Salute San Raffaele, Milan, Italy; ^16^Unit of Immunology, Rheumatology, Allergy and Rare Diseases, IRCCS Ospedale San Raffaele, Milan, Italy; ^17^Department of Internal Medicine, Pneumonology, Allergology and Clinical Immunology, Central Clinical Hospital of the Ministry of National Defense, Military Institute of Medicine, National Research Institute, Warsaw, Poland; ^18^Department of Autoimmune Diseases, Institut d’Investigacions Biomèdiques August Pi I Sunyer (IDIBAPS), Hospital Clínic of Barcelona (ERN-RITA Center), University of Barcelona, Barcelona, Spain; ^19^Autoimmune and Rare Diseases Unit, Department of Internal Medicine, Hospital Universitario Ramón y Cajal, Universidad de Alcalá de Henares, IRYCIS, Madrid, Spain; ^20^Department of Rheumatology, “Evangelismos” General Hospital, Athens, Greece; ^21^Department of Pediatric Rheumatology, Faculty of Medicine, Hacettepe University, Ankara, Türkiye; ^22^Division of Rheumatology, Department of Internal Medicine, Faculty of Medicine, Marmara University, Istanbul, Türkiye; ^23^Unit of Hematology, Azienda Ospedaliera Universitaria Senese and University of Siena, Siena, Italy; ^24^Bioengineering and Biomedical Data Science Lab, Department of Medical Biotechnologies, University of Siena, Siena, Italy; ^25^Ophthalmology Unit, Department of Medicine, Surgery and Neurosciences, University of Siena, Siena, Italy; ^26^Department of Medical and Surgical Sciences (DIMEC), University of Bologna, IRCCS Azienda Ospedaliero-University of Bologna, Bologna, Italy

**Keywords:** AIDA Network, Castleman’s disease, epidemiology, interleukin-6, international registry, prognosis treatment

## Abstract

Castleman’s disease (CD) consists of a wide spectrum of rare disorders classified into unicentric CD and multicentric CD (MCD), based on the diffusion of disease distribution and the severity of clinical manifestations. While unicentric CD is characterized by a single lymph node involvement, MCD is defined by multiple lymph node station involvement with more prominent systemic symptoms. MCD is further subdivided into HHV-8 associated MCD, polyneuropathy, organomegaly, endocrinopathy, monoclonal plasma cell disorder, skin changes (POEMS)-associated MCD, and idiopathic MCD (iMCD), which is also subdivided into iMCD-TAFRO (thrombocytopenia, anasarca, fever, reticulin fibrosis, organomegaly) and iMCD-NOS (not otherwise specified). The rarity of the disease makes it still poorly understood, as current insight is largely based on case reports and relatively small patient cohorts. Therefore, knowledge about the clinical details of the disease, histological correlations, complications, prognostic factors, and optimal treatment management remains incomplete. The potential offered by the creation of online data sharing makes the development of a registry specifically dedicated to CD a necessary step to conduct solid research on this condition. Building on the experience and widespread international reach of the AutoInflammatory Disease Alliance (AIDA) Network, the development of this registry can allow the recruitment of a sufficient number of patients to conduct robust research in all the fields of the disease. Moreover, the AIDA Network itself will enable multidisciplinary and integrated collaboration among the various figures necessary for the optimal diagnostic, clinical, and therapeutic management of patients affected by CD in its different forms.

## Introduction

Castleman’s disease (CD) refers to a group of rare disorders that predominantly impact the lymph nodes. It is classified into unicentric CD and multicentric CD (MCD), depending on the clinical presentation and the extent of disease involvement. In particular, unicentric CD is characterized by a single lymph node involvement with mild systemic symptoms that are usually successfully treated by surgically removing the affected lymph node. Conversely, MCD is defined by multiple lymph node station involvement with more prominent systemic symptoms. MCD is further subdivided into HHV-8 associated MCD (which can be either HIV-negative or HIV-positive), polyneuropathy, organomegaly, endocrinopathy, monoclonal plasma cell disorder, skin changes (POEMS)-associated MCD, and idiopathic MCD (iMCD), which is also subdivided into iMCD-TAFRO (thrombocytopenia, anasarca, fever, reticulin fibrosis, organomegaly) and iMCD-NOS (not otherwise specified) (1).

The diagnosis of Castleman’s disease requires a lymph node biopsy with CD-defining histological features. For iMCD-NOS diagnosis, the enlarged lymph nodes must be in ≥2 lymph node stations; in addition, at least two clinical and/or laboratory abnormalities must be present and exclusion of mimickers of the disease must be assessed, as for the diagnostic criteria proposed by Fajgenbaum et al. (2). Different criteria are applied to diagnose iMCD-TAFRO, but a lymph node showing characteristics of CD is still required (3).

Based on a randomized, double-blind, placebo-controlled trial, siltuximab, a chimeric monoclonal antibody that binds to interleukin-6, is the main therapeutic option for iMCD (4). Actually, a *post hoc* analysis of the phase 2 siltuximab trial found that progression-free survival was significantly improved in patients treated with siltuximab compared to those receiving a placebo (5). However, in this trial approximately one-third (34%) of patients responded to the drug, highlighting the heterogeneity of treatment response and the need for further clinical investigation.

Despite advances in classification and therapy, several unmet needs remain regarding the natural history, prognosis, and management of CD subtypes, particularly in idiopathic and rare forms. Comprehensive, multicenter data are essential to better characterize the disease spectrum and inform evidence-based care.

The Autoinflammatory Disease Alliance (AIDA) is an international network that links various physicians specializing in rare autoinflammatory disorders and inflammatory eye diseases. One of the main objectives of the AIDA Network is to develop disease registries dedicated to rare inflammatory diseases, and to date it has already developed and launched 10 registries which have made a significant scientific contribution in a short period (6–14). Indeed, the number of rare autoinflammatory diseases is continuously expanding, increasingly involving diverse and innovative aspects of the innate immune system, including actinopathies, interferonopathies, and NF-κB-mediated disorders (15, 16). For more details, the AIDA Network may be accessed at the following website: https://aidanetwork.org/en/.

Given the potential offered by the internet, the creation of online registries has become an essential tool for conducting research based on a sufficient number of patients in the field of rare diseases. Indeed, the implementation of rare disease registries is also recommended by the European Union Committee as a means of collecting enough data for epidemiological and clinical research (17–19). Therefore, promoting a registry dedicated to a rare and poorly diagnosed disease like CD is both desirable and necessary. To date, there is no international prospective registry designed to collect standardized, real-world data specifically on Castleman’s disease across its entire clinical spectrum.

Various systemic diseases can mimic CD, such as autoimmune, autoinflammatory diseases and vasculitic disorders (2, 20, 21). Consequently, different physicians are closely involved with CD, including pathologists, haematologists, internists, immunologists and rheumatologists. As a result, a collaboration between these figures is essential and the development of an international Network is desirable to shed new light to this condition.

Based on the experience gained over the years by the AIDA Network in the field of registries dedicated to rare diseases, an additional international patient registry specifically dedicated to CD has been developed. This registry aims to collect longitudinal, real-world data from a large cohort of patients with CD, with the ultimate goal of improving disease characterization and clinical management.

The primary objective of this study is to describe the rationale, structure, and implementation process of the International AIDA Network registry dedicated to CD, aiming to support future clinical and research efforts in the field.

## Materials and methods

### Study design

The AIDA registry for CD has been established as an international, physician-led, electronic-based registry dedicated to patients diagnosed with all forms of Castleman’s disease.

Patient data collection consists of a retrospective phase, covering information gathered up to the time of enrollment in the registry, and a prospective phase, which includes ongoing data collection after enrollment. The prospective phase requires at least one follow-up visit per year; however, updates should be made whenever there is a change in treatment strategy and/or the occurrence of a complication.

The registry is designed to collect demographic, clinical, laboratory, and treatment-related data starting from disease onset. No additional information will be required beyond the standard assessments performed as part of routine follow-up. Consequently, no extra costs or financial burdens will be incurred. Likewise, participation in this initiative will not impact therapeutic decisions. Data elements were selected based on a comprehensive literature review. Specifically, studies indexed in PubMed, Scopus, and Web of Science up to 31 January 2024 were reviewed using keywords such as “Castleman disease,” “iMCD,” “TAFRO,” and “POEMS.” Variables were chosen in collaboration with clinical experts, particularly haematologists and rheumatologists.

The registry is open to centers and specialists worldwide involved in the diagnosis, treatment, and management of Castleman’s disease. Centers interested in participating can join the AIDA Network by reaching out to the Promoter, contacting the AIDA Team via email at support@aidaregistry.org, or completing a dedicated form available at: https://aidanetwork.org/en/aida.

A key requirement for inclusion in this project is obtaining approval from the local ethics committee. Additionally, each center must designate a principal investigator to oversee local coordination of the study and at least one site investigator responsible for managing documentation and data collection. Upon formally expressing the intention to participate in the International AIDA Network registry for Castleman’s disease, both the principal investigator and the site investigator will receive credentials to access the registry and begin patient enrollment.

The project is overseen by an international scientific committee composed of representatives from participating centers, which reviews the scientific coherence of the registry and evaluates data usage proposals. In addition, a central coordinating unit based at the lead institution, together with local investigators, is responsible for ensuring data quality. Quality control mechanisms include initial and ongoing training for investigators responsible for data entry, use of guided data entry procedures with mandatory fields, periodic checks for consistency and completeness, and randomized audits of submitted cases.

A minimal core dataset has been defined to ensure consistent and high-quality data collection across all participating sites. This dataset includes key variables such as demographic information, diagnostic criteria, histologic features, clinical subtypes, laboratory findings, and treatment data. These fields must be filled in for all patients enrolled; in this regard, periodic checks will be performed to identify and flag incomplete or inconsistent entries. Standardized definitions and data dictionaries are distributed through the study protocol to all centers. Furthermore, training sessions are provided upon the inclusion of each new site within the AIDA Network to promote harmonization in data entry. Regular centralized monitoring ensures data accuracy and uniformity across the registry.

As for the project timeline, the enrolment phase is currently expected to last 10 years, with the possibility of extension in the future; nevertheless, interim analyses will be conducted based on the specific objectives and research hypotheses of each study aim, as soon as a sufficient cohort of patients is available to ensure meaningful statistical analysis.

Authorship will be granted to all contributors who have made substantial contributions to the study design, data collection, data analysis, or manuscript preparation. The number of authors from each center will be proportional to the number of patients enrolled by that center in the registry and to the total number of patients included in the study sample.

External collaborations are welcome, provided they are communicated to and approved by the AIDA Network coordination. Proposals will be evaluated based on their scientific relevance, alignment with the project’s objectives, and adherence to the registry’s governance policies.

### Registry objectives

The primary aim of the registry is to collect data on the largest possible number of patients affected by CD, in order to enable clinical research focused on epidemiology, a better understanding of the disease’s clinical features, including its unusual and atypical presentations, as well as the diagnostic and therapeutic aspects requiring further development. Secondary aims of the registry are: to achieve the following research objectives: (i) to determine more thoroughly the epidemiology of the disease, specifically assessing any differences in incidence by sex, age, ethnicity and geographical areas; (ii) to identify specific subgroups of patients who have akin clinical and laboratory characteristics; (iii) to better correlate the different clinical phenotypes with their respective histological features; (iv) to update the international diagnostic criteria and to establish whether they have to be adapted into classification criteria; (v) to develop criteria to monitor disease activity; (vi) to describe the different therapeutic options in terms of response to treatment and duration of sustained efficacy as well as to investigate the optimal timing for tapering medication and, if tapering is not feasible, to determine the statistically significant minimal effective schedules; (vii) to understand differences in response to therapy between the TAFRO and non-TAFRO disease variant and the therapeutic outcome in patients with POEMS; (viii) to identify strategies for treating patients who experience secondary loss of efficacy; (ix) to assess if cycling different therapies can restore effectiveness to best standard of care; (x) to determine predictive factors for treatment response; (xi) to characterize complications such as malignancies, to both identify predictive factors for their development and to establish whether treatment reduces the risk of their occurrence; (xii) to gather any specific details on bone marrow characteristics and peripheral blood smear in CD patients; (xiii) to measure the disease’s impact on quality of life; (xiv) to evaluate the influence of socioeconomic status on healthcare access and the effects of the disease on patient absenteeism and presenteeism; (xv) to evaluate the survival rate and the causes of death, as well as how these are influenced by different therapeutic approaches; and (xvi) to identify any prognostic factors.

Table 1 summarises the objectives of this registry.

**Table 1:** This table describes the primary and secondary objectives of the registry dedicated to Castleman’s disease.

Primary objective	To gather information in a real-world context on as large as number of patients affected by CD as possible
Secondary research objectives	To determine the epidemiology of the disease, specifically regarding differences in incidence by sex, age, ethnicity and in distinct geographical areas
	To identify specific subgroups of patients with akin clinical and laboratory characteristics
	To correlate the different clinical types of CD with their respective histological features
	To update the international diagnostic criteria and eventually adapt them into classification criteria
	To develop criteria to monitor disease activity
	To describe the different therapeutic options in terms of response to treatment and assessment of efficacy over time; to investigate the optimal timing for tapering medication and, if tapering is not feasible, to determine the minimal effective dose or schedule
	To understand differences in response to therapy between the TAFRO and non-TAFRO disease variants as well as the therapeutic outcome in patients with POEMS
	To identify strategies for treating patients who experience secondary loss of efficacy
	To evaluate whether cycling different therapeutic strategies can restore effectiveness to best standard of care
	To search for possible predictive factors to treatment response
	To characterize complications such as malignancies in CD to both identify any prognostic factors for their development and to establish whether treatment may reduce the risk of their occurrence
	To gather any specific details on bone marrow characteristics and peripheral blood smear that could help discern the disease
	To measure the diseases’ impact on quality of life
	To evaluate the influence of socioeconomic status on healthcare access and the effects of the disease on patient absenteeism and presenteeism
	To evaluate the survival rate and the causes of death, as well as how these are influenced by different therapeutic approaches
	To search for prognostic factors related to long-term survival and the occurrence of disease complications

CD, Castleman’s disease; POEMS: polyneuropathy, organomegaly, endocrinopathy, monoclonal plasma cell disorder, skin changes; TAFRO, thrombocytopenia, anasarca, fever, reticulin fibrosis, organomegaly.

### Inclusion/exclusion criteria

Both patients with unicentric and multicentric CD may be included in this registry. All patients enrolled must have a lymph node biopsy that histologically confirms the diagnosis of Castleman’s disease. To further distinguish between iMCD-NOS, iMCD-TAFRO, and iMCD-POEMS, the corresponding diagnostic criteria must be applied (3, 22). To fulfil these criteria, mimicking haematological, rheumatological, and infectious diseases should be ruled out.

Eligibility criteria will be applied by local investigators based on the fulfilment of the specific sets of criteria, where applicable (2, 3, 22). In cases with overlapping clinical or histopathological features, including mimicking conditions such as IgG4-related disease, autoimmune disorders, or lymphomas, inclusion will be allowed only if diagnostic criteria will be met. Ambiguous or borderline cases will be discussed with expert panels to ensure consistent interpretation of criteria and avoid misclassification.

Before participating, patients must give their written informed consent after receiving a comprehensive explanation of the project. This includes an overview of the registry’s objectives, assurance that their involvement will not affect their medical care or treatment decisions, their unrestricted right to withdraw consent at any moment, and details on the legal safeguards in place to protect their privacy, anonymity, and data security. Patients must be explicitly informed that their decision to participate or decline will in no way impact their medical treatment. Additionally, they have the right to request the permanent removal of any personal data previously recorded in the registry, as long as they formally submit their request to the study promoter (University of Siena).

If a patient lacks the capacity to give consent, permission must be secured from their legally appointed representative, who is responsible for ensuring compliance with the study protocols until participation ends or consent is revoked. Additionally, for individuals aged 12 and above, their explicit agreement is a prerequisite. All data are collected through an online platform.

For data collection and storage, the study utilizes the Research Electronic Data Capture (REDCap) system, a secure digital platform developed by Vanderbilt University Medical Center (VUMC) (23). Specifically designed to support the administration of online surveys and databases, REDCap streamlines data management while ensuring reliability and security.

The registry can be accessed at: https://aidanetwork.org/en/register/castleman-disease. All collected data are securely housed on the University of Siena’s servers in Siena, Italy. To ensure confidentiality, principal and site investigators are restricted from viewing data gathered by other participating centers.

Researchers are responsible for meticulously inputting data into the online registry and maintaining its accuracy. The principal investigator must validate the correctness of all submitted records. Access to the system is securely regulated through individual login credentials, ensuring strict confidentiality and robust data protection.

To facilitate global participation and reduce language barriers, the data entry interface is provided solely in English.

### Ethics

In June 2019, the Ethics Committee of the Azienda Ospedaliero-Universitaria Senese in Siena, Italy (Ref. No. 14951), issued the first national regulatory approval for the AIDA Project.

Patient data management adheres to the EU General Data Protection Regulation (GDPR) (2016/679/EU) or equivalent regulations in other regions, ensuring compliance with data protection and privacy standards (24). As for voluntary informed consent, the AIDA registries follow the ethical principles outlined in the Declaration of Helsinki.

The data collected in the registry are centralized and managed in compliance with current data protection regulations. Researchers within the AIDA Network may submit analysis proposals, which will be evaluated by a scientific committee. Access to the data is subject to project approval and adherence to shared ethical and methodological criteria.

Neither patients nor physicians receive any financial compensation for participating in the study. Additionally, there are no agreements for billing with national healthcare systems or insurance providers.

### Statistical analysis

The statistical analyses will be designed according to the specific characteristics and type of data being processed, ensuring alignment with the objectives of studies carried out within the AIDA Network. Therefore, given the heterogeneous nature of the research questions addressed, a variety of statistical approaches will be applied, ranging from descriptive statistics and hypothesis testing to advanced multivariate models, according to the nature of the outcomes and study designs involved. Both principal investigators and site investigators are encouraged to present their research proposals at AIDA meetings. Data gathered at a single center can be analyzed independently by other satellite centers, provided that the AIDA Network is properly acknowledged in any resulting publications. Meanwhile, the entire dataset collected through the registry will be overseen by a team of statisticians and physicians within the network, selected for their specialized expertise.

Missing data will initially be addressed through a descriptive analysis of their distribution and, if necessary, by applying multiple imputation techniques under the assumption that data are missing at random (MAR).

## Results

**Figure 1:**
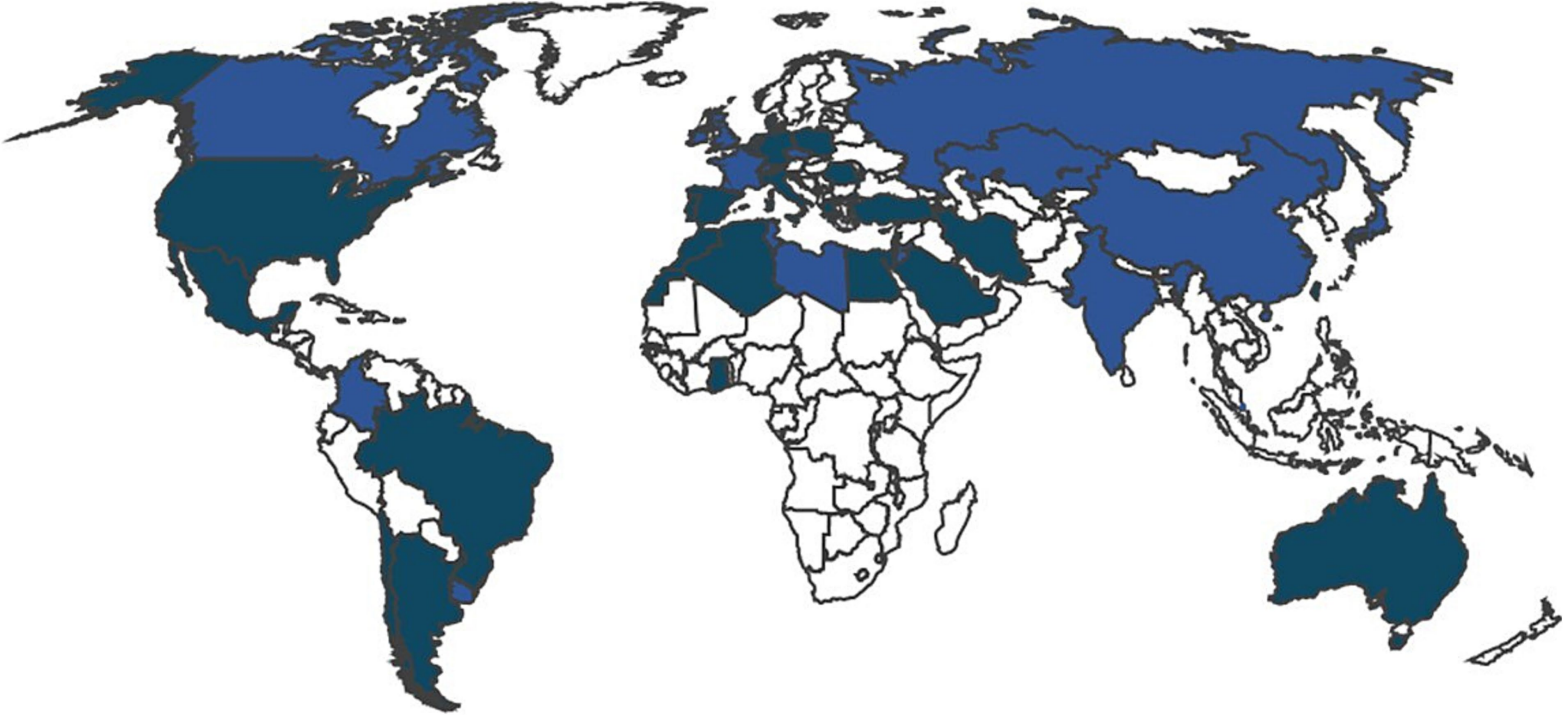
Worldwide distribution of the AIDA Network. The figure highlights in blue the countries covered by the network across the various continents.

To date, 45 nations in 4 continents (Algeria, Argentina, Australia, Belgium, Brazil, Canada, Chile, China, Colombia, Cyprus, Czechia, Denmark, Dominican Republic, Egypt, France, Germany, Ghana, Greece, India, Iran, Ireland, Israel, Italy, Japan, Jordan, Kazakhstan, Lebanon, Libya, Morocco, Mexico, Netherlands, Norway, Poland, Portugal, Romania, Russia, Saudi Arabia, Singapore, Slovenia, Spain, Switzerland, Taiwan, Tunisia, Turkey, United States) have already joined the AIDA Network. The Figure 1 highlights the current (January 2025) worldwide distribution of the AIDA Network.

The AIDA project has seen participation from 273 centers worldwide, involving a total of 785 users, including 273 principal investigators, 512 site investigators, 1 lead investigator, and 4 data managers. This project is officially registered on ClinicalTrials.gov (ID: NCT05200715).

### Registry development

The registry is intended to gather data on Castleman’s disease through both retrospective and prospective approaches, with an emphasis on the patient’s clinical, laboratory, and treatment history.

As of January 2024, the registry holds 3,013 standardized data elements, each corresponding to a specific variable for the study, divided into 11 separate instruments. Table 2 provides a comprehensive overview of these instruments, detailing their phases and the total number of fields they contain.

**Table 2:** Instruments constituting the registry dedicated to patient with Castleman’s disease; the number of fields and at which phase of data collection they are referred to is also reported.

Instruments	Fields	Retrospective/Prospective phase	No. of mandatory fields
Demographics	11	Retrospective phase	4
Consents	5	Retrospective phase	2
General information about Castleman’s disease onset	7	Retrospective phase	
Histopathology	10	Retrospective phase	0
Diagnosis	36	Retrospective phase	0
Castleman’s disease features up to the enrolment	229	Retrospective phase	0
Laboratory data during disease activity	49	Retrospective phase	0
Treatment strategies attempted over time	3	Retrospective phase	2
Treatments performed up to the time of the enrolment	1,160	Retrospective phase	0
Follow-up visits-the prospective phase	1,488	Prospective phase	41
Death of the patient (to open only in case of patient’s death)	4	Retrospective/prospective phase	0

**Figure 2:**
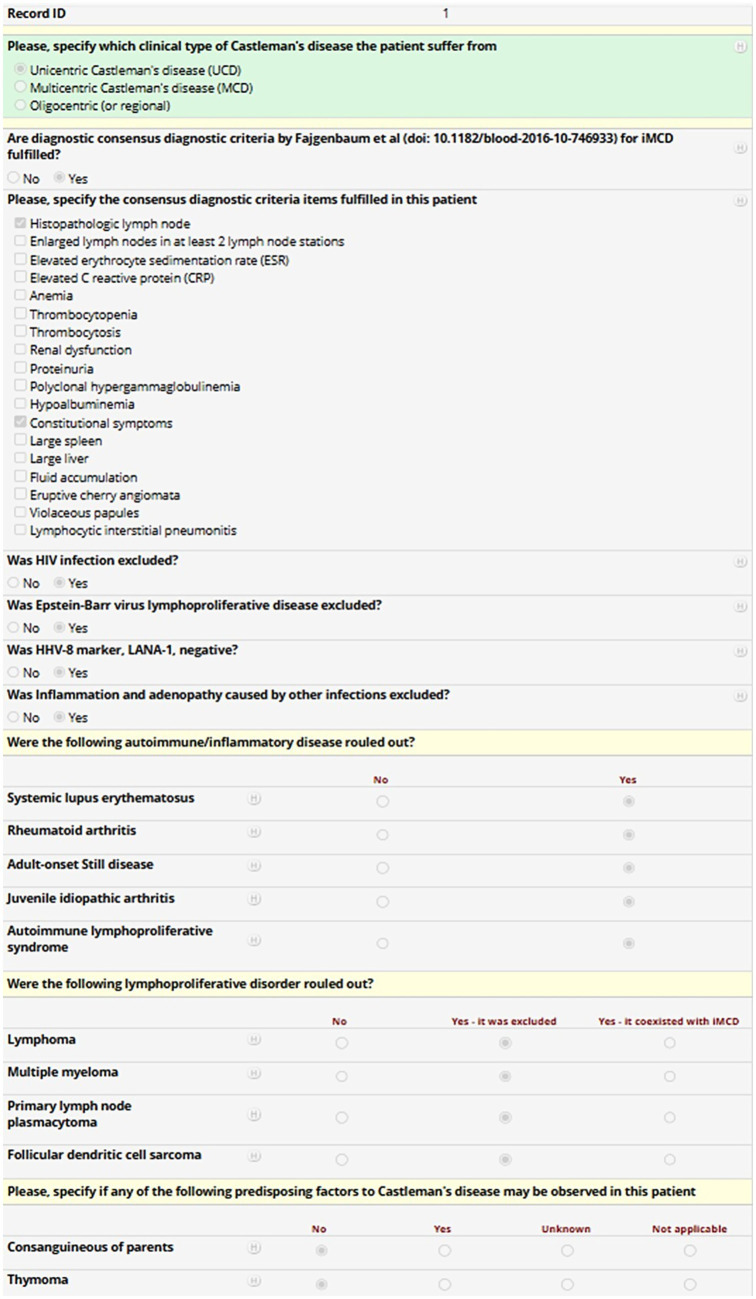
Mock patient entry screen from the REDCap-based International AIDA registry dedicated to Castleman disease. The screenshot shows a representative data entry form for a fictional patient. The form includes clinical variables about the type of Castleman disease, the fulfilment of diagnostic criteria for idiopathic multicentric Castleman’s disease, the exclusion of infectious, autoimmune or neoplastic mimickers, and the identification of predisposing factors, with field types such as checkboxes and radio buttons. Conditional logic (“branching logic”) is applied throughout the form. For instance, in the case of multicentric Castleman’s disease, the investigator may select the appropriate subtype or indicate fulfilment of specific diagnostic criteria. This structure enables streamlined, adaptive data collection while reducing unnecessary fields and improving usability and data quality.

Each variable is required solely when it applies to the patient’s clinical profile, managed through an adaptive branching logic system. This system dynamically triggers additional fields only when necessary, based on the information already provided. As a result, investigators will only be prompted to complete the relevant subset of variables, ensuring a streamlined and tailored data entry process. Figure 2 provides a representative screenshot of the REDCap interface used in the International AIDA registry dedicated to Castleman disease, aimed at demonstrating the structure of clinical data fields and branching logic applied to streamline data entry.

## Discussion

Castleman’s disease is a rare disorder that requires a multidisciplinary approach for diagnosis and management. The epidemiology of the disease is poorly understood, with varying prevalence reported in Japan and the United States (7, 25). Moreover, the incidence of iMCD across sexes has not yet been clearly established (26–28). Developing an international registry that includes data from multiple continents and countries would enhance our understanding of the disease’s epidemiology.

As mentioned previously, a lymph node showing histological features consistent with CD is mandatory for diagnosis. The three histological variants of CD are the hypervascular variant, plasma cell variant, and mixed variant. UCD and iMCD-TAFRO are more frequently associated with the hypervascular variant, whereas iMCD-NOS, HHV-8-MCD, and POEMS-MCD are more often linked to the plasma cell or mixed variants (1). However, these histological features do not aid in distinguishing between disease subtypes and do not correlate with treatment response.

In terms of diagnosis, several diseases must be excluded, including haematological conditions (e.g., lymphoma, multiple myeloma) and rheumatological disorders. For the latter, iMCD-NOS frequently presents with arthritis, making it challenging to differentiate from other rheumatological conditions that also present with constitutional symptoms, arthritis or arthralgia, and lymphadenopathy. Examples include Still’s disease, systemic lupus erythematosus, IgG4-related disease, and the recently described VEXAS syndrome. The diagnostic challenge is further compounded by the fact that histological analyses of enlarged lymph nodes in these disorders can show Castleman-like features (23, 29–32). Consequently, identifying more specific characteristics and/or biomarkers of the disease and refining diagnostic criteria could improve diagnostic accuracy. The current diagnostic criteria for iMCD may require validation considering the most recent findings on CD (2). The major criteria may potentially exclude patients with atypical or incomplete or not yet fully developed forms of CD, while the minor criteria appear to be extremely non-specific and largely shared with diseases that need to be differentiated from CD. Consequently, it would be useful to validate the criteria based on a large population with no geographical or ethnic constraints. Regarding therapeutic options, treatment strategies vary depending on the subtype of iMCD. POEMS-MCD is treated according to standard POEMS protocols (33), while iMCD-NOS and iMCD-TAFRO are generally managed similarly. Assessing the differences in treatment response among these subclasses would be highly beneficial. Siltuximab is the only approved therapy for iMCD-NOS and iMCD-TAFRO; however, only about one-third of patients experience significant symptom improvement (4). Rituximab is another useful therapy, particularly in non-severe cases. In severe forms that are unresponsive to IL-6 inhibition, chemotherapy is advised (34). Alternative regimens, such as mTOR inhibitors (sirolimus), anti-IL1 agents (primarily anakinra), JAK inhibitors (ruxolitinib), proteasome inhibitors (bortezomib), lenalidomide/thalidomide, and cyclosporin A, are supported only by case series and reports (35–48).

Overall, the optimal management of patients who develop secondary therapy inefficacy is not well understood. Research is required to determine whether alternative therapies can restore responsiveness to standard care and whether employing cyclic therapies could effectively lead to a regain of efficacy in therapies previously burdened by loss of efficacy. It also remains unclear whether tapering treatment regimens is possible and, if so, in which subset of patients. Therefore, further research is needed to evaluate the efficacy, duration of remission, predictive factors for response to treatments, and how to manage treatment in terms of tapering and the utilization of different molecule rotation.

Patients with MCD have a higher risk of developing malignancies (49). Prognostic factors for malignancy development have not yet been identified, and it remains unknown whether current therapies reduce this risk. Also, it is crucial to investigate survival rates and causes of death among both treated and untreated patients. These elements will be better investigated thanks to the development of this registry.

Noteworthy, some diagnostic limitations are inherently associated with CD itself and may affect data collection within the registry. These include the rarity of the disease, overlapping clinical features with mimicker diseases such as autoimmune and lymphoproliferative disorders, and the need for histological confirmation, which may not always be uniformly available across centers. To address these issues, the registry has been designed to request, where available, the application of validated diagnostic criteria for iMCD, iMCD-TAFRO, and POEMS-MCD, in line with current international recommendations. This approach will help promote consistency and allow for stratified analyses based on diagnostic certainty.

## Conclusion

In conclusion, Castleman’s disease is a complex condition that necessitates a multidisciplinary approach for accurate diagnosis and management. Ongoing efforts are essential to better understand the most effective ways to treat patients with the different forms of the disease.

The international scope and widespread reach of the AIDA project can facilitate the recruitment of a sufficient number of patients to conduct robust research, while the network itself will enable multidisciplinary and integrated collaboration among the various figures necessary for the optimal diagnostic, clinical, and therapeutic management of patients affected by CD in its different forms.

## Data Availability

The original contributions presented in the study are included in the article/supplementary material, further inquiries can be directed to the corresponding authors.
